# The Odyssey of Integration: Is Management its Achilles’ Heel?

**DOI:** 10.5334/ijic.5440

**Published:** 2020-02-26

**Authors:** Robin Miller, K. Viktoria Stein

**Affiliations:** 1 University of Birmingham, UK; 2 International Foundation for Integrated Care, UK

**Keywords:** integrated care, management, competences, culture, leadership

## Abstract

**Introduction:**

The importance of management to the implementation of integrated care is recognised in evidence and practice. Despite this recognition, there is a lack of clarity about what ‘good’ management of integrated care looks like, if the competences are different to management for ‘traditional’ care, and how such competences can be acquired.

**Theory and methods:**

This exploratory study is based on qualitative interviews with participants with extensive experience of implementing integrated care in senior professional, research, administrative and/or policy roles. It conceptualises management as working at ‘strategic’ and ‘operational’ levels.

**Results:**

Management of integrated care was seen to require an ability to create networks across professions and organisations, to be comfortable with distributing responsibilities, and to thoroughly understand the wider system. Competences to support these new ways of working included an understanding of how to implement people-centredness, to have courage to challenge the status quo, and to demonstrate humility to learn from others. Structured development opportunities for managers were lacking, but seen as vital for the sustainability of change.

**Discussion and conclusion:**

Management for integrated care remains an underdeveloped concept and practice. A first formulation of the competences necessary was achieved, but more work is urgently required to understand how to better prepare and support managers to achieve necessary changes in practice and culture.

## Introduction

Reviews of evidence consistently highlight management as either an enabler or barrier to successful implementation of integrated care [1,2,3,4,54]. This includes management at both strategic and operational levels [5]. Leadership was traditionally conceptualised as primarily focussed on those in senior positions but more recent thinking emphasises distributed leadership across multiple management levels [6,7], and indeed the contribution of other stakeholders such as clinicians and those with lived experience [8,54,55]. Supportive and engaged leadership by managers is seen to make a positive contribution but neglect or resistance by leaders present major risks to the achievement of successful outcomes [1,4,9,10,11,12,13,54]. Evaluations of individual programmes also reflect the importance of proactive management to successful implementation [e.g. 14,15,16,17]. Contributions of strategic managers that have been identified to date are bringing together stakeholders, mobilizing action within organisations by emotionally connecting individuals with their vision, securing internal and external resources for implementation including to support learning, fostering a culture of innovation, and prioritising involvement of communities within strategic developments [12,13,15,17,54]. Operational managers’ contribution includes generating confidence within frontline staff of their ability to implement, communicating the overall vision in a version that is understandable by the frontline, quantifying tangible benefits for service recipients, and practical coordination of activities and resources [1,4,16]. In addition to specific management functions, many of the general enablers of integrated care fall wholly or largely under the remit of managers. These include shared patient records, pooling of budgets, team-based processes, co-location, partnership governance, opportunities for development, person-centric quality improvement, and introduction of new roles [4,10,16,17,18,19]. Such enablers commonly require both strategic and operational management within specific management functions such as finance, human resources, information technology.

Whilst its importance is clear, the nuances of what ‘good’ management for integrated care involves is less understood. Much research essentially describes supportive local contexts in which those in key management roles are willing to commit personal and other resources to facilitate more integrated care, and are able to trust that colleagues in other agencies will not behave opportunistically. The behaviours that support such contexts, and how it can be encouraged and developed in areas which are not fortunate to have established a positive collaboration are however unclear. The extent to which strategic and operational management of integrated care is different to management of “traditional care”, and if existing approaches to developing competences amongst managers is sufficient, are similarly unknown. This then creates an ‘Achilles heel’ for those embarking on the long journey to more integrated care – with the right management capacity, competence and commitment the connected challenges can be overcome, but all too often these are not in place leading to a well conceived programme not being well implemented and sustained beyond an initial pilot phase. Similarly, attempts to diffuse innovations beyond the areas with the initial champions for change flounder when they fail to find receptive and competent management contexts.

This exploratory study seeks to respond to these important gaps in our knowledge about the management of integrated care. It is based on interviews with those with extensive experience of developing and implementing collaborative approaches within clinical, strategic, operational and/or policy roles at a regional, national and international level. Those with direct experience of providing or facilitating the voice of those who have accessed health and care services are also included. It considers three main questions – is strategic and operational management of integrated care different to that of traditional care, what competences are connected with the management of integrated care, and how can we support the development of such competences in practice. At the outset, the study specifically addressed management, purposefully excluding the term leadership, as it was the intention to focus on those who have to make integrated care work on a daily basis.

## Methodology and Theory

### Theoretical background

The question of what makes a good manager has interested philosophers and statesmen throughout the ages. Whilst there is recognition that the demands made on a manager will vary dependant on the context and their particular role, considerable work has been done to clarify what competences are required by managers within healthcare settings [13,18,28,30,31,49,56]. Pihlainen et al (2016)’s [57] systematic review suggests that competences related to the healthcare context have social, organisational, and financial dimensions, and that these should be demonsrated alongside operational (i.e. quality improvement, credibility with professionals, and staff development) and general management (i.e. strategic mindset, interpersonal skills and time management). Likewise the question of what constitutes the difference between management and leadership. “Scientific” management theories started to be developed at the beginning of the 20th century, the most famous of the early works being Fredrerick Winslow Taylor’s “The Principles of Scientific Management” [20]. Other notable works include McLelland’s Human Motivation Theory [e.g. 21,22] or Kotter’s leadership versus management challenge [e.g. 23 and later].

In the field of integrated care however, this discussion has only recently emerged, as a result of the questions on how to scale, transfer and sustain change in a complex environment. Whilst frameworks underline the importance of the management function in integrated care [e.g. 18,24], these do not specify the elements of what constitutes good management for integrated care [e.g. 25,54,55]. There is emerging evidence of how difficult it is to manage both the change process and a resultant integrated care system [26,27,55]. Leadership, and in particular ‘systems leadership’, is often portrayed as the dynamic solution to overcome long-standing barriers, with management being relegated to the less appealing role of administering what leaders have achieved [7]. That said, there is no consensus even on whether leadership and management in integrated care are two distinct concepts, or merely two sides of the same coin [28].

For the purposes of this study and to answer the research questions outlined above, Mintzberg’s distinction between strategic (Strategic Apex) and operational management (Middle Line) was selected as the basis for discussion during the interviews and the analysis of the results [29,30]. This approach was selected, because this study aimed at understanding management better, and Mintzberg does not classify leadership as an opposing or separate concept to management. Indeed, Mintzberg went on to say, that “[I]n place of heroic leaders who don’t manage, health care needs engaged managers who lead.” [31, p. 190] The description of the two levels of management were thus adapted to suit the needs of the interviews [based on 29,30,32]:

**Strategic Managers** – decision makers, interaction with environment, setting policies, goals & frameworks, supervision of operational managers.

**Operational Managers** – implementers, mediators between strategic and core, limited interaction with environment related to their delivery areas, departmental strategies within strategic framework.

Managers responsible for integrated care may be doing so within the boundaries of a single organization, in a distinct partnership role connected with two or more organisations, or of hybrid roles that incorporate both single and partnership responsibilities. All of these were included in the study as they all require managers to introduce new ways of working, to deploy resources differently, and to achieve person centred outcomes in order to provide more integrated care. In the results and the discussion sections, the appropriateness of this approach as well as the necessity to distinguish between management and leadership will be explored in more detail.

### Methodology

In order to understand what makes a good manager of integrated care, and answer the research questions, an exploratory, qualitative approach was chosen in form of in-depth interviews. A purposive sample of managers and reseachers of integrated care was put together, covering North America, Europe and Australasia. Interviewees were selected based on their long-standing experience on both levels and in various roles (policy, practice, person/caregiver, research) over at least 10 years in the integrated care field. Overall 50 people were invited to take part in the study, 25 of whom accepted the invitation, and two declined. Ultimately, 22 interviews were conducted by RM and VS, lasting between 25 and 74 minutes. The interview guidance can be found in Appendix 1. The interviewees represented 13 different countries from four continents, and four supra-national or international organisations (see Table 1). There was parity of gender amongst participants. The interviews were conducted either online or in person by one of the study authors, and were recorded and transcribed by a professional verbatim.

**Table 1:** Characteristics of participants by roles/stakeholder group.

	Female/male	North America	Europe	Western Pacific Region	Internal organisations*	Policy	Practice	Person/Caregiver	Research

Interviewee 1	F		✓			✓	✓	✓	✓
Interviewee 2	M			✓		✓	✓		✓
Interviewee 3	M		✓				✓		✓
Interviewee 4	M		✓			✓	✓		✓
Interviewee 5	M			✓		✓	✓		✓
Interviewee 6	M				✓	✓			✓
Interviewee 7	F		✓			✓			✓
Interviewee 8	M		✓			✓	✓		✓
Interviewee 9	F	✓				✓	✓	✓	✓
Interviewee 10	F		✓			✓	✓		✓
Interviewee 11	F				✓	✓			
Interviewee 12	F		✓		✓	✓		✓	✓
Interviewee 13	F	✓						✓	
Interviewee 14	M			✓		✓			✓
Interviewee 15	M				✓	✓			
Interviewee 16	M	✓					✓		
Interviewee 17	M		✓			✓	✓		✓
Interviewee 18	M	✓				✓	✓		✓
Interviewee 19	F			✓			✓		✓
Interviewee 20	M		✓				✓		
Interviewee 21	F			✓					✓
Interviewee 22	M				✓	✓			✓

* International organisations encompass UN agencies, Bretton Woods Institutes, European Union, and transnational NGOs.

Many respondents represented more than one perspective, which reflected their long-standing and diverse careers in integrated care. Geographic representation was skewed towards Europe, because most known, published and internationally active experts still work there. While the authors of the study are well aware that there are many experts and initiatives in Latin America and Africa, who would have been equally qualified, accessibility, time and language restrictions were the main barrier to inclusion.

The transcripts were analysed and coded separately by each study author. The codes were then discussed and consensus was reached as to the final coding framework [33]. The coding was performed on the full text statements and only fill words or repetitions were left out. The coding framework is described in Table 2. Initial codes were derived directly from the interview questions, and were subsequently altered, deleted or renamed, where it was found necessary and after discussion between the two study authors. A paucity in quotes did not automatically lead to a deletion of the code, as it was seen as an interesting outcome in itself. The originator of quotes within the findings are identified through a number relating to Table 1.

**Table 2:** Coding framework.

Code name	References overall	No of interviews referencing code

Developing competences	111	18
Strategic competences	59	15
Operational competences	23	13
Context	53	15
Culture	41	11
Personal commitment and resilience	17	12
Community perspective and holistic understanding	22	9
Doctors and management	29	9
Management vs leadership	27	10
Other issues	48	11
Good examples	20	10
Bad examples	9	4
Stakeholders	4	4

Ethics approval was granted by the Humanities and Social Sciences Ethical Review Committee at the University of Birmingham (ERN_18-1347).

## Findings

### Management of Integrated Care

Participants expressed varied opinions as to the extent to which management of integrated care was different. For some, it was essentially standard practice deployed in a particular context. Unsuccessful implementation was due to a lack of commitment by operational and/or strategic managers or that they were not competent managers of usual or integrated care. Most participants however did see management of integration as requiring different ways of understanding and behaving as a strategic or operational manager compared to ‘traditional care’. These include an ability to focus on societal not just organisational value, to work towards holistic and not just financial performance targets, to be comfortable with distributed and not solely hierarchical and linear approaches, a willingness to challenge and not be comfortable with the status quo, and meaningful not tokenistic public involvement. In addition, there was general agreement that the need for an understanding of the wider system was crucial, but rarely found in managers.

And first I thought maybe there’s no big difference, so you have to fight your same battles if you are normal manager in a hospital and …but the difference is that you are more on a change management role and you really have to have the skills of a change manager…and endurance to knock on every door again and again and again and at the tenth time it will maybe open a little bit. (Interviewee 3)

The Mintzberg distinction between operational and strategic was seen as helpful although the roles were often re-labeled as ‘senior’ (strategic) and ‘middle’ (operational) management. Leadership was interpreted by some participants as primarily being the responsibility of those in more senior, strategic positions but most reflected Mintzberg’s perspective [30,31,32] that integrated care requires leadership to be demonstrated at all levels as part of management responsibilities. The emphasis on distribution of such influence was articulated by one participant as a move from ‘leader*ship* roles’ to ‘leader*ful* systems’ [34]. There was also a noticeable tendency for inspirational individuals to being singled out as a main catalyst of integrated care becoming a local or national priority and reality. This did not go as far or as gendered as stating that the development was due to a ‘great man’ but did suggest that one outstanding person had been central to generating momentum within the local context.

Strategic managers were described as crucial to new visions of integrated care being developed and structural legitimacy being gained through formal governance structures within providers, funders and government. Strategic managers engaging directly with people and communities made symbolic and practical differences. Practical in that their views were seen more likely to have influence if it had been heard in the first person, and symbolic in that it demonstrated to others within organisations that this was seen as a valuable contribution. Furthermore experiencing authentic listening and responsive action by strategic managers encouraged public representatives to continue their involvement

You see her [vice-president] everywhere. If you saw her in the waiting room, she might be sitting with someone, you might think she’s a care giver or she’s a patient herself, but she wants to get to know her clients and there’s a genuineness about her that people really relate to and she’s very, very caring. I see her with her subordinates who are not maybe pulling their weight and she really encourages them to do that more with a carrot rather than with a stick. I imagine that she wouldn’t put up with someone who is not doing that for very long. (Interviewee 13)

Once the vision is set, operational managers are instrumental to the hard work of implementation including overcoming active resistance to the connected changes and general inertia to any form of transformation. Strategic managers supported through providing ‘permission’ for operational managers to be entrepreneurial in their recognition of opportunities and mobilising the necessary resources to unlock the connected value. Culture was mentioned by many participants as the historical partnership context in which integration was being introduced and a relational dynamic which required active intervention. Both levels had responsibility to role model new practices and constantly emphasise the rationale and vision of integrated care. Operational managers maintained implementation momentum using influence, inspiration and more directive means to ensure professionals and others adopt the expected behaviours:

They follow up and monitor or they push, they fight, they push again, they require from their staff or the supervisors, so that the overall management role is very important because there is always a need for somebody to actually push forward for the change. (Interviewee 18)

The dynamic between managers and clinicians is well recognised as an enabler or barrier of quality in health care. According to participants clinicians, and in particular doctors, appeared something of a mixed blessing to achieving more integrated care. All recognised the influence of the medical professional regarding implementation with several particpants reporting the major contribution that an inspirational doctor had made to progressing more person-centred care. Using the credibility based on their expertise and contact with patients, they had challenged the status quo maintained by managers to encourage or force it to be more flexible and responsive.

Whereas the people who’ve been instrumental in leading the change initially have been clinical leaders locally and yet they wouldn’t necessarily perceive to be…to have a management role per se. (Interviewee 17)

Others had experienced a less positive contribution in which clinicians were not open to major changes being made by managers to traditional arrangements in which they felt comfortable professionally and personally. This resulted in clinicians, and in particular doctors, acting as principle resistors to a more integrated care. There was similar polarisation of views as to the ability of doctors to take on management roles – with some believing that their professional paradigms and skills precluded them from being competent managers, whils others reporting that hybrids who could combine clinical competence and credibility with management were the most influential and impactful:

Clinical people are good at individual one on one: diagnosis, treatment, coaching, encouraging and so forth. Management have to think across boundaries at scale about systems. It’s an almost exactly opposite skillset. And healthcare is full of management people who used to be clinical people. In our system we pretty much have outlawed that. (Interviewee 12)

So I think then to call it out clearly, the doctors are probably the group that are struggling most with the idea of integrated care, because they have to give away. (Interviewee 8)

I would argue that there shouldn’t be a rule that you don’t appoint clinicians. I have a left-hand, right-hand law. What we are good at is the right hand, what we are bad at is the left hand, but many doctors use both hands. (Interviewee 18)

Most supported the powerful impact of doctors, and indeed other clinicians and practitioners, working collaboratively with managers to achieve shared leadership within an organisation or system.

the contribution of both in terms of mutually reinforcing dynamic is really important I think. And one cannot function without the other in my experience on that journey. (Interviewee 17)

### Competences for management

Competences are comprised of “K(nowledge), S(kills)s, A(ttitudes) [which] are the abilities and characteristics that enable a job holder to accomplish the activities described in a task statement that describes what the job holder does.” In other words, it’s about “what I do, what I can do and how, and why I do it.” [35] While teaching knowledge and skills is straight forward and widely practiced, attitudes cannot be tought directly, but are influenced by teachers, superiors, mentors and peers [36].

There was general agreement amongst participants that in order to be a successful manager of integrated care, both aspects, learned behaviour and innate personality, were important, thus echoing the discussions in more recent management theory that both intrinsic and extrinsic factors make good managers [56,58]. Whilst some managers had a more natural leaning towards integrated care, it was thought that all could develop the necessary competences with personal commitment and a supportive environment. Furthermore, even those with more innate abilities benefitted from formal and informal development opportunities.

I think the personality traits are largely intrinsic, but I think some of the technical skills around pushing the boundaries are teachable because we get them from experience, so through our experiential learning and through our apprenticeship in the system…. And we would need to give consideration to how we support those attributes emerge and develop, because I think it is developing them rather than building them from scratch. (Interviewee 2)

Using the knowledge, skills and attitudes concept of competences as a basis, the interviews were analysed to extract which competencies were identified as crucial for integrated care managers to be successful. A summary of the identified competences with a description and illustrative quote can be found in Table 3. Participants were not able to articulate specific differences in the competences of operational and strategic managers so they are presented as a ‘generic’ set. This is further supported by the general agreement that the competences themselves wouldn’t necessarily change, rather the scale and scope as well as the personal ability to perform grew over time and with changing roles.

**Table 3:** Knowledge, Skills and Attitudes for Managers of Integrated Care.

	Explanation	Quote

**Knowledge**		

*Technical knowledge*	This includes professional knowledge; management theory and theories of innovation; understanding of the workings of the overall system (and not just their own organization or sector); being able to understand social determinants of health; and being able to apply knowledge in the context of integrated care.	“the knowledge that she had from the actual needs and the actual how things operate was probably critical to navigate the landscape and then … she had knowledge of the political setting and how policy works and she managed to play very well between those two fields and make something that is a compromise that fits both sides.” (Interviewee 19)
**Skills**		

*Taking a people-centred approach*	Involving patients, people and communities in every step of the way; using their perspective as the guiding principle in the design, implementation and evaluation of care; challenging existing power bases which could diminish the importance of lived experiences; engaging communities whose voice is often unheard due to disadvantage and exclusion	“a the fundamental rethink is they’re the managers. They manage their care, they coordinate the team, not that a patient or family has to do all those but we have to flip on its head the notion of who we work for. And we work for people and their families.” (Interviewee 9)
*Communication*	Communicating through alternative means and formats, including social media in order to engage a broader range of stakeholders; framing opportunities in paradigms and language that will be relevant to different professional and sectorial groups; ability of listening actively, being present during a conversation; creating the time to go out and listen to people.	“you have to check in so many times to make sure that what you have either said or communicated has been received in the way that you thought it was being communicated, because I have found that most of the time it’s not and I have been completely gobsmacked by how differently people have – even from what I have said and what I have communicated – they have received something completely different, a completely different message than the one that I was sending out and I find that absolutely fascinating.” (Interviewee 1)
*Building and maintaining relationships*	Bringing people together who previously had little contact and/or experienced tensions; fostering a supportive and trusting environment; creating the space for new solutions to emerge; building networks; understanding relationship management as an integral and ongoing task.	“…because I’ve realised that you could have all the intellectual prowess you want but if you don’t know how to help people and teams and integrated teams and cross-organisations and systems thrive, you can’t integrate care. It’s all relational. So I think it has to be taught in every aspect of every curriculum.” (Interviewee 9)
*Distributive leadership and collaborative approach to management*	Being able to let go; trusting your team; creating the conditions; managing by shared outcomes; managing teams across professions and organisations; giving permission to innovate and be flexible.	“…something requiring tolerance, ambiguity, organic iterative and allowing people latitude to be able to do stuff and one of the things that’s emerged strongly is the tension between central control and command at the centre of organisations, … just as letting go of that control and trusting to people who know their business and empowering them to do stuff and when you do that, that tends to work out well.” (Interviewee 17)
*Being a coach and a mentor*	Developing and implementing inter-disciplinary CPD programmes; giving constructive feedback; understanding management as a coaching role rather than a ‘command and control’ role.	“So I think that issue about how do we invest, not just in the formation but actually in the development. And I think that’s about giving people opportunities to learn, to share, to innovate, to fail and to hopefully move on and do things better.” (Interviewee 5)
*Managing culture change*	Identifying culture as a key topic to be managed; understanding different cultures and culture change; managing culture as a continuous work in progress.	“This is a big culture change and you don’t find people and you don’t change people easily to a completely different culture than the one that they signed up for in the first place… you tend to revert to the average or revert to the norm if you don’t continue, to continually renew the new reality and that’s hard work, because we’re trying to do something that’s not just a little bit different but something that’s significantly different from the norm.” (Interviewee 12)
**Attitudes**		

Resilience	Being able to weather the storm; be persistent; withstand constant resistance; being able to cope with loneliness.	“you really have to have the skills of a change manager and you need I think a lot of resilience and endurance to knock on every door again and again and again and a tenth time it will maybe open a little bit.” (Interviewee 16)
Courage	Standing up against the norm; addressing uncomfortable truths; approaching people and organisations, which are opposing the change.	“I think that’s a really important part of encouraging people to be brave in integrated care, so if you think of the characteristics of a good chief executive, you know, there’s all of that high EQ stuff, there’s all of the competencies that come with leadership, but one of those competencies is bravery and bravery is not just about being stuck on your idea, it’s about being brave enough to fail and to learn the lessons of those failures…, so that characteristic of teaching flexibility …is something that can be taught and that comes through in that experiential learning.” (Interviewee 2)
Humility	Accepting mistakes and learning from them; helping others to thrive; taking another persons’ perspective; being able to see the big picture.	“And the ones that I’m attracted to in terms of a role are the people who are reflective about the failure and can understand the multidimensional aspects of that and, most importantly, understand their role in that failure. So did they listen enough, did they codesign enough, did they stay in the problem long enough. How did they deal with it when it was all going wrong, did they offer the resignation or did they go and say ‘mea culpa’ and then what was their response.” (Interviewee 2)

Technical knowledge was considered as the basis for any good manager. Building on the individuals’ professional background, an in-depth knowledge of integrated care theories, frameworks, models and tools was considered a given, along with knowledge of management, leadership, innovation and systems. An interesting element of learning was highlighted by one interviewee stating that professionals had already a lot of knowledge, but did not know how to apply it in the new context or how to connect the dots. Many interviewees acknowledged that they had never really received any training and some even mentioned that they only found out afterwards, that what they were trying to do actually had a name.

The skills necessary to manage integrated care successfully all relate to people and relationship management in essence. As one interviewee put it: “*Soft skills are actually the hard skills*.” All participants agreed that communication was one crucial skill for success. There was general recognition that communication was hard work, did not receive enough attention and had to be targeted towards different audiences and different levels. Taking a people-centred approach was identified as a skill, rather than an attitude, as it reinforced and underpinned many management tasks related to integrated care. This categorisation also emphasised the conviction of the participants, that a people-centred approach was teachable and needed to be implemented actively.

Integrated care is a means to overcome sectoral barriers and break down professional prejudices, so being able to reach out across professions and organisations to establish and maintain a strong network of providers, include the target population or community, and create trusted relationships was identified as essential for sustainable change.

The skill of distributive leadership and having a collaborative approach to management reflects the difficulty of participants to clearly distinguish between management and leadership. It was clearly identified as a skill, however, as it was considered teachable, especially through continuous development programmes and action learning. The importance of continuous learning was emphasised throughout the interviews and reflected in the need for a manager to be a coach and teacher, supporting the professional and personal development of staff and colleagues.

Managing culture (and culture change) was unanimously considered a vital and separate skill, which many managers tend to ignore in the beginning of their endeavours, to often detrimental effect. In an organisational context, individual attitudes translate into collective professional and organisational cultures – if personal attitudes and the culture of the environment don’t overlap, a conflict will arise. As integrated care usually challenges established cultures, managing this conflict was identified as a key competence.

Several participants also emphasised the importance of personal adjectives such as being brave, stubborn, persistant, resilient, and having a fire or passion burning inside. It was acknowledged that not everyone was cut out to be a manager for integrated care, as it meant dealing with a lot of stress, pressure and uncertainty, as well as being attacked from the ‘top’ (e.g. policy makers and funders) and from the ‘bottom’ (e.g. operational staff and clinicians) if expectations were not met in short timescales and anxieties were running high due to changes in established practices and cultures. The right attitude included representing core values, embracing the community and patients as key partners on a daily basis, being approachable, accessible and being seen. Other issues raised were around the ethics of integrated care, legal requirements, the lack of integration between research and practice towards a more improvement-based management approach or the size of organisations.

### Development of competences

The respondents had a very diverse background, many having filled different roles and worked in various sectors over time, and none of them had straight forward career paths (see also Table 1). The majority had previous experience in service delivery, and had then moved on to management positions within organisations and/or the public sector. A combination of academia and practice was not unusual, nor was the involvement in policymaking and private enterprises. This also meant that most interviewees had changed organisations several times, often citing the search for more meaningful and impactful ways of changing the system, personal development and curiosity of learning the “other sides’ viewpoint” as the reason. Questioned about their background in management and integrated care, they reveiled a piecemeal education and training experience, some of them seeking very specific training through MBA programmes, others preferring a learning by doing approach. But all agreed that current programmes were not enough, and that there were not sufficient opportunities to hone the competences outlined above. There was also consensus that while making one’s own mistakes was necessary, many of the pitfalls, which lead to failure or stalling of an integrated care initiative, could and should be avoided through better education, training and preparation of managers. General agreement was also found in the conviction that integrated care principles should be part of every training programme, and not limited to health and social care or a specialist MBA course. In regard to preparing for the future and proactively identifying managers for integrated care, these competences should also be included in Continuing Professional Development (CPD) programmes.

Another key message was the call for recognising management of integrated care as a job or role in and of itself, which needed to be rewarded appropriately. It was not something one could do on top of a day job or on the fly, as so often happens in practice. By giving such responsibilities proper recognition in relation to capacity and status, it would also make it easier to connect with peers and build a peer network. There was a desire to create such a network for support, peer learning, mentoring and feedback, as a means to deal with the stress and pressure mentioned throughout the interviews. This was found to be especially necessary as managing integrated care often meant a dual role within ones’ own organisation and as part of a wider integrated care network. Conflicts arising through split loyalities and accountibilities added to the existing pressure, especially when the role within the integrated care initiative did not match executive power in one’s own organisation. While this might seem a challenge on the senior level, it was actually reported on all levels, e.g. managing a care team across different sectors and organisations, or facilitating the steering committee of an integrated care alliance. The set of comptences to deal with this conflict reinforced previous statements around role clarity, communication and networking skills, as well as resilience. Many interviewees would have wished to have a peer support network at hand to reach out to for advice and support.

Putting these two elements, structured training programmes combined with peer learning, together, the ideal approach to developing the competencies necessary to manage integrated care should include a strong focus on case-based learning with the possibility of learning on the job. Ultimately, the goal of managing integrated care should be to design a learning organisation, which can not only react and adapt to the continuously changing environment, but influence and co-design it, together with its partners. As a manager, this requires a skillset around how to build and maintain learning environments, how to use monitoring and evaluation as a positive influencer to reinforce the necessity for change, how to establish a continuous professional development programme within the organisation, and how to reassure and empower professionals in the face of these changes.

[integrated care programmes] are innovative and they require from all staff, from low level staff to highest managers and policy makers even, to think about service delivery or their role in what they do differently. Managers set the tone, set the directions, support and strengthen the changes which are not always easy. Make sure that those changes are actually happening so they follow up and monitor or they push, they fight, they push again, they require from their staff or the supervisors. (Interviewee 18)

## Discussion

The question of whether there was a clear difference between competencies for operational and strategic managers was not answered conclusively as many found it difficult to differentiate between the two. While some participants acknowledged that they were useful concepts to structure thinking and discussion, others challenged the whole notion as being set in the current hierharchical system and thinking, and that it needed new technical terms as well as new concepts to describe these roles of the future. One participant went so far as to call for a new language to describe integrated care to capture the innate difference to the status quo. There was also a definitive tendency to see these roles in the context of the system the managers were working in, and as part of a learning journey for managers who gradually progress from more junior to senior roles. Inherently the distinction was made between for example team managers, who would have the oversight of several teams and have a more operational role of implementation, and the system managers, who were seen to have a distinctive policy role, reaching out to stakeholders and providing the vision and framework for integrated care. There was agreement that the competences remained the same, and the question was more around the degree and maturity of the knowledge, skills and attitudes acquired, which made the difference. This ambiguity is reflected in the literature, where management usually is not recognised as a task in itself, but implicated in other activities, which lead to successful integration like change management, quality improvement, communication, organisational culture and leadership, or managing teams [13,24,37,38,39,40,41,54].

Similary, Stein (2016) identified the necessity of building competences on all levels of the system as a building block for sustainable integrated care [42].

There were interesting parallels with research regarding the management of networks. In some ways this is inevitable, as networks are a recognised approach to achieving more integrated care [59,60]. Indeed, local integration often begins through voluntary networks in which clinicians, people with lived experience and/or managers come together to respond to the needs of a particular geographic or other community [61]. Such networks can be instrumental to governments deciding to form integrated care policies through highlighting the inequalities of access, outcomes and experience caused by fragmentation, and/or to local services delivering practical solutions to improve care within their locality [62]. A challenge for government is often how to replicate the benefits of local voluntary networks in areas in which they have not emerged informally, as mandation can stifle the reciprocity and intrinsic motivations that contribute to the original progress [63]. Network managers are crucial to the success of voluntary and mandated networks but have to encourage engagement and coordinate activity of members with little of the traditional power bases available to them [64]. Success is instead reliant on their ability to develop a shared identity and purpose, to demonstrate that the network will respond to existing incentives and challenges experienced by partners, and to facilitate opportunities for building relationships and trust [65]. Such management work has been termed ‘orchestration’ and been described as the 11^th^ management role to the ten roles initially outlined by Mintzerg [66,67]. The emphasis on ‘soft skills’, distributing responsibilities, effective communication, and ability to cope with emerging flexibilities mirror the competences which were connected with integrated care managers [68,69]. The integrated care manager has to become a boundary spanner [65], who has the power and comptences to overcome conflict, create shared values and collective accountability, and drive disruptive innovation.

The importance of leadership in developing initial support and common vision for integrated care, and inspiring professionals and managers to work more collaboratively is again confirmed [8,43]. The issue of who is best placed to provide this leadership, particularly in localities in which it does not emerge through existing individuals or networks, remains unresolved. Professionals and managers working together to lead integrated care should present as a powerful coalition as they bring different expertise and legitimacies, but there remains the potential for turf-wars over who should be the most influential (‘*the* leader’) and a danger that those with less influence, such as people with lived experience and in low paid roles, are excluded from such allegiances [44,45]. Furthermore managers have to learn how to engage with new sets of ‘professional’ leaders who may have different perspectives and incentives to those with a health setting. Work to articulate the competences or characteristics of ‘leaders’ within integrated care systems reflects what was found in this study for managers [eg 43,46] – this potentially suggests that development opportunities for managers in relation to integrated care could usefully be extended to those within other roles. An acknowledgement that the process of integration is collaborative, recursive and ongoing, and requires managers to contiuously work on establishing, revising and reinforcing the purpose, relationships, and trust between all partners reflects findings in network theory [70]. Also confirmed is the need for managers to implement the vision of leaders through undertaking ‘the graft’ that accompanies any transformation programme [47].

Systems and organisations need to move towards a networked, learning environment, which emphasises co-design, shared responsibilities and an active and continuous approach to learning on the job. So rather than distinguishing between strategic and operational, or manager and leader, the emphasis should be to understand the competences for integrated care and then teaching and living them as part of the culture and vision for integrated care. With experience, these competences are reinforced and thus enable people to move to different levels and roles. So far, no competency framework for integrated care exists, let alone one for managers of integrated care. However, Langins and Borgermans (2015) [48] made a significant first attempt and identified five competency clusters for the health workforce in integrated care. These encompassed patient advocacy, effective communication, team work, people-centred care, and continuous learning – all of which were reflected in the competences identified in the current study. The “Leadership Competencies for Healthcare Services Managers” [49] blow in a similar horn with communication and relationship management, professional and social responsibility, health and healthcare environment, business, and leadership as the five domains of competences for managers. The significant impact of a strategic and coordinated approach to continuous education and training of all involved was also recognised by extending the Triple Aim and adding the improvement of the work-life balance of staff to the concept [50]. The present exploratory study synthesises all these different frameworks and experiences to identify key competences for managers of integrated care.

There is another underlying challenge buried in this discussion and that is the realisation that we are trying to capture new concepts with old technical terms. There is a conflict between the call for shared decisionmaking, shared outcomes and shared accountability, which are then to be implemented and managed by medical directors for integrated care or chief executive officers. The struggle to maintain change and build sustainable integrated care systems is also a struggle between the old and the new power, where the former is held by a few and represented by hierarchies and the latter is held by many and represented by networked organisations [51]. The solution to this struggle may be a recognition that both hierarchy and networks will be required to ensure that dynamic innovations can emerge, but there is sufficient stability and order for these to be implemented consistently [70]. This will however require a common set of values to be adopted, in which those with structural power are willing to concede existing arrangements to new ideas [52,53]. Managers will undoubtedly have an important role in achieving such synergies.

## Limitations

There are several limitations to this study, which reflect its exploratory nature. First of all, while there was every effort made to have a balanced representation of all stakeholders and geographic regions, the respondents primarily reflect the clinical-managerial tradition of North America, Europe, Australia and New Zealand. This was partly due to the reach of the network, but also to language and time restrictions. All interviews were conducted in English, which required not only a high level of command of the language, but also the confidence to express oneself in technical terms. Some respondents, specifically from civil society organisations and non-English native speakers, did not consider themselves capable or ‘expert enough’ to participate in the interviews. Secondly, due to the time consuming nature of the study, not all initially scheduled interviews could be conducted and the number of interviews was limited. This may have lead to the omission of competences or aspects of managing integrated care, which could have shed more light on the complex nature of the task at hand. Thirdly, there has not been triangulation to provide validity that the perspectives of participants regarding their own management reflects the reality experienced by others. Finally, the findings have not been validated yet, but preliminary findings were presented to a workshop at the 19th International Confernce for Integrated Care in San Sebastian, and the feedback informed the finalisation of the study.

## Conclusion

In the myth of Achilles, the baby is dipped in the river Styx to make him invulnerable, but is held by the heel which is therefore not protected. Whilst integrated care programmes contain many vulnerabilities, this research supports our contention that management, and a lack of recognition of and investment in appropriate skills and capacity, remains a consistent weakness. The role has so far received little attention in theory or practice having rather been taken for granted. Internationally there is a common failure to articulate what is different about such management, to actively prepare people to undertake such roles, and to recognise the unique challenges that they face. Further research is needed to understand what is required in general for those managing integrated care, and if there is variation connected to practice contexts, stages of implementation, forms of integration and regional location. The current gaps in knowledge present a considerable vulnerability to the mainstreaming of integrated care as managers are central to embedding new cultures, processes and alliances. There is an urgent need to actively identify, support and train future managers, and to ensure that those already in such roles have time for reflection and learning. A competency framework for integrated care management would help to guide development opportunities, and have potential value within recruitment processes. This should form part of a wider organisational and cultural shift towards the development of learning environments, which build upon the strengths of individuals and foster personal and professional development. Such a movement would also support the necessary shift towards more distributed and equitable management, which is inclusive of individuals, communities and “auxiliary” professions. Above and beyond a conducive systems and organisational context, this study has brought to light that resilience, humility and “an inner fire” are paramount for managers of integrated care to weather the storms and persevere in their endeavours to change not only one organisation, but systems, cultures, and attitudes. It is time to acknowledge their crucial role in taking integrated care to the next level and giving the support necessary to make this change happen.

## Additional File

The additional file for this article can be found as follows:

10.5334/ijic.5440.s1 Appendix 1. Management and leadership competency interview guide.

## References

[1] Cameron A, Lart R, Bostock L, Coomber C. Factors that promote and hinder joint and integrated working between health and social care services: a review of research literature. Health Soc Care Community, 2014 5; 22(3): 225–33. DOI: 10.1111/hsc.12057 23750908

[2] Borgermans L, Marchal Y, Busetto L, Kalseth J, Kasteng F, Suija K, Oona M, Tigova O, Rösenmuller M, Devroey D. How to improve integrated care for people with chronic conditions: Key findings from EU FP-7 Project INTEGRATE and beyond. International journal of integrated care, 2017 7; 17(4). DOI: 10.5334/ijic.3096 PMC5854097 29588630

[3] Kadu MK, Stolee P. Facilitators and barriers of implementing the chronic care model in primary care: a systematic review. BMC Fam Pract, 2015 2 6; 16(12). DOI: 10.1186/s12875-014-0219-0 PMC4340610 25655401

[4] van Duijn S, Zonneveld N, Montero AL, Minkman M, Nies H. Service integration across sectors in Europe: Literature and practice. Int J Integr Care, 2018 Apr-Jun; 18(2): 6 DOI: 10.5334/ijic.3107 PMC6095054 30127690

[5] Mintzberg H. The Fall and Rise of Strategic Planning. Harvard Business Review, Jan-Feb 1994.

[6] Gronn P. Distributed leadership as a unit of analysis. The Leadership Quarterly, 2002; 13(4): 423–451. DOI: 10.1016/S1048-9843(02)00120-0

[7] Fillingham D, Weir B. System leadership: lessons and learning from AQuA’s integrated care discovery communities London: King’s Fund; 10 2014 https://www.kingsfund.org.uk/sites/default/files/field/field_publication_file/system-leadership-october-2014.pdf.

[8] Evans JM, Daub S, Goldhar J, Wojtak A, Purbhoo D. Leading integrated health and social care systems: Perspectives from research and practice. Healthc Q, 2016; 18(4): 30–5. DOI: 10.12927/hcq.2016.24553 27009705

[9] Auschra C. Barriers to the integration of care in inter-organisational settings: a literature review. International Journal of Integrated Care, 2018; 18(1): 5 DOI: 10.5334/ijic.3068 PMC5887071 29632455

[10] Busetto L, Luijkx K, Calciolari S, Ortiz LGG, Vrijhoef HJM. Barriers and facilitators to workforce changes in integrated care. International Journal of Integrated Care, 2018; 18(2): 17 DOI: 10.5334/ijic.3587 30127701 PMC6095071

[11] González-Ortiz LG, Calciolari S, Goodwin N, Stein V. The core dimensions of integrated care: A literature review to support the development of a comprehensive framework for implementing integrated care. Int J Integr Care, 2018 8 8; 18(3): 10 DOI: 10.5334/ijic.4198 PMC6137610 30220893

[12] WHO 2015. People-centred and integrated health services: an overview of the evidence Geneva: World Health Organisation; 2015 https://www.who.int/servicedeliverysafety/areas/people-centred-care/evidence-overview/en/.

[13] Suter E, Oelke ND, Adair CE, Armitage GD. Ten Key Principles for Successful Health Systems Integration. Healthc Q, 2009; 13(Spec No): 16–23. DOI: 10.12927/hcq.2009.21092 20057244 PMC3004930

[14] RAND Europe, Ernst & Young LLP. National Evaluation of the DH Integrated Care Pilots. Rand Health Quarterly, 2012; 2(1): 8 https://www.rand.org/pubs/periodicals/health-quarterly/issues/v2/n1/08.html. 10.7249/rhq-02-01-08 PMC4945290 28083230

[15] Nolte E, Frølich A, Hildebrandt H, Pimperl A, Schulpen GJ, Vrijhoef HJ. Implementing integrated care: A synthesis of experiences in three European countries. International Journal of Care Coordination, 2016; 19(1–2): 5–19. DOI: 10.1177/2053434516655626

[16] Miller R. Transforming Integration through General Practice: Learning from a UK Primary Care Improvement Programme. Int J Integr Care, 2018 Apr-Jun; 18(2): 13 DOI: 10.5334/ijic.3044 PMC6095053 30127697

[17] Nicholson C, Hepworth J, Burridge L, Marley J, Jackson C. Translating the elements of health governance for integrated care from theory to practice: a case study approach. Int J Integr Care, 2018 1 31; 18(1): 11 DOI: 10.5334/ijic.3106 PMC5854213 29588645

[18] Minkman M. The Development Model for Integrated Care: a validated tool for evaluation and development. Journal of Integrated Care, 2016; 24(1): 38–52. DOI: 10.1108/JICA-01-2016-0005

[19] Borgermans L, Devroey D. A Policy Guide on Integrated Care (PGIC): Lessons Learned from EU Project INTEGRATE and Beyond. International journal of integrated care, 2017 7; 17(4). DOI: 10.5334/ijic.3295 PMC5854173 29588631

[20] Taylor FW. The Principles of Scientific Management New York: Harper and Brothers; 1911.

[21] McClelland DC. The Two Faces of Power. Journal of International Affairs, 1970; 24(1): 29–47.

[22] McClelland DC, Boyatzis RE. Leadership Motive Pattern and Long Term Success in Managament. Journal of Applied Psychology, 1982; 67: 737–43. DOI: 10.1037/0021-9010.67.6.737

[23] Kotter JP. A Force for Change How Leadership differs from Management. New York: The Free Press; 1990.

[24] Valentijn PP, Boesveld IC, Van der Klauw DM, Ruwaard D, Struijs JN, Molema JJ, Bruijnzeels MA, Vrijhoef HJ. Towards a taxonomy for integrated care: a mixed-methods study. International Journal of Integrated Care, 2015; 15(1): None. DOI: 10.5334/ijic.1513 PMC4353214 25759607

[25] WHO Europe. EUR/RC66/15 Strengthening people-centred health systems in the WHO European Region: framework for action on integrated health services delivery Copenhagen: WHO Regional Office for Europe; 2016 http://www.euro.who.int/en/health-topics/Health-systems/pages/publications/2016/eurrc6615-strengthening-people-centred-health-systems-in-the-who-european-region-framework-for-action-on-integrated-health-services-delivery.

[26] Bengoa R, Stout A, Scott B, McAlinden M, Taylor MA. Systems, not Structures: Changing Health & Social Care. Expert Panel Report for the Ministry of Health of Northern Ireland. Belfast; 2016 Accessed 26 August 2019. https://www.health-ni.gov.uk/publications/systems-not-structures-changing-health-and-social-care-full-report.

[27] Ham C, Walsh N. Making integrated care happen at scale and pace: Lessons from experience London: The King’s Fund; 2013 https://www.kingsfund.org.uk/publications/making-integrated-care-happen-scale-and-pace.

[28] Goodwin N. Change management In Amelung VE, Stein KV, Goodwin N, Balicer R, Nolte E, Suter E (eds.). Handbook Integrated Care. New York: Springer Nature; 2017 DOI: 10.1007/978-3-319-56103-5_1

[29] Mintzberg H. Structure in fives. Designing Effective Organizations. Prentice Hall; 1983.

[30] Mintzberg H. The Manager’s Job: Folklore and Fact. Harvard Business Review; 1990 Mar-Apr.

[31] Mintzberg H. Managing the Myths of Health Care Bridging the Seperations between Care, Cure, Control, and Community. Oakland: Berrett-Koehler Publishers 2017 DOI: 10.1007/978-3-319-53600-2_1

[32] Mintzberg H. Mintzberg on Management Inside Our Strange World of Organizations. New York: The Free Press; 1989 DOI: 10.1007/978-1-349-20317-8_23

[33] Gale NK, Heath G, Cameron E, Rashid S, Redwood S. Using the framework method for the analysis of qualitative data in multi-disciplinary health research. BMC Med Res Methodol, 2013 9 18; 13: 117 DOI: 10.1186/1471-2288-13-117 24047204 PMC3848812

[34] Raelin J. From leadership-as-practice to leaderful practice. Leadership, 2011; 7(2): 195–211. DOI: 10.1177/1742715010394808

[35] Quiñones MA, Ehrenstein A. Editors. Training for a rapidly changing workplace: Applications of psychological research Washington, DC: American Psychological Association; 1997 DOI: 10.1037/10260-000

[36] McClelland DC. Testing for competence rather than for intelligence. American Psychologist, 1973; 28: 1–14. DOI: 10.1037/h0034092 4684069

[37] Gilburt H. Supporting integration through new roles and working across boundaries London: The King’s Fund; June 2016 https://www.kingsfund.org.uk/sites/default/files/field/field_publication_file/Supporting_integration_web.pdf.

[38] WHO Europe. Lessons from transforming health services delivery: compendium of initiatives in the WHO European Region Copenhagen: WHO Regional Office for Europe; 2016 http://www.euro.who.int/__data/assets/pdf_file/0014/303026/Compendium-of-initiatives-in-the-WHO-European-Region-rev1.pdf?ua=1.

[39] Goodwin N, Dixon A, Anderson G, Wodchis W. Providing integrated care for older people with complex needs Lessons from seven international case studies. London: The King’s Fund; 1 2014 https://www.kingsfund.org.uk/publications/providing-integrated-care-older-people-complex-needs.

[40] Jelphs K, Dickinson H, Miller R. Working in Teams Bristol: Policy Press; 2016 DOI: 10.2307/j.ctt1t88zd0

[41] Minkman MMN, Vermeulen RP, Ahaus KTB, Huijsman R. A survey study to validate a four phases development model for integrated care in the Netherlands. BMC Health Services Research, 2013; 13: 214 DOI: 10.1186/1472-6963-13-214 23758963 PMC3733829

[42] Stein KV. Developing a Competent Workforce for Integrated Health and Social Care: What Does It Take? International Journal of Integrated Care, 2016; 16(4): 9 DOI: 10.5334/ijic.2533 PMC5354207 28316549

[43] Social Care Institute for Excellence (scie). Leadership in integrated care systems: Report prepared for the NHS Leadership Academy Future of care. London: Social Care Institute for Excellence; 11 2018: 9 Accessed 13 September 2019. https://www.scie.org.uk/integrated-care/leadership/systems.

[44] Dickinson H, Ham C, Snelling I, Spurgeon P. Are we there yet? Models of medical leadership and their effectiveness: an exploratory study Final report. NIHR Service Delivery and Organisation Programme; 2013 http://www.netscc.ac.uk/hsdr/files/project/SDO_FR_08-1808-236_V07.pdf.

[45] Batalden M, Batalden P, Margolis P, Seid M, Armstrong G, Opipari-Arrigan L, Hartung H. Coproduction of healthcare service. BMJ Qual Saf, 2016 7; 25(7): 509–17. DOI: 10.1136/bmjqs-2015-004315 PMC4941163 26376674

[46] Ghate D, Lewis J, Welbourn D. Systems leadership: exceptional leadership for exceptional times Synthesis Paper. London: Virtual Staff College, The Colebrooke Centre for evidence and implementation; 10 2013 http://www.cevi.org.uk/docs/Systems_Leadership_Synthesis_Paper.pdf.

[47] Dickinson H. Making a reality of integration: less science, more craft and graft. Journal of Integrated Care, 2014 12 15; 22(5/6): 189–96. DOI: 10.1108/JICA-08-2014-0033

[48] Langins M, Borgermans L. Competent health workforce for the provision of coordinated/integrated health services Working Document. Copenhagen: WHO Regional Office for Europe; 2015 http://www.euro.who.int/__data/assets/pdf_file/0010/288253/HWF-Competencies-Paper-160915-final.pdf.

[49] International Hospital Federation. Leadership Competencies for Healthcare Services Managers Geneva: International Hospital Federation; 2015 https://www.ihf-fih.org/resources/pdf/Leadership_Competencies_for_Healthcare_Services_Managers.pdf.

[50] Bodenheimer T, Sinsky C. From Triple to Quadruple Aim: Care of the Patient Requires Care of the Provider. Ann Fam Med, 2014; 12: 573–576. DOI: 10.1370/afm.1713 25384822 PMC4226781

[51] Heimans J, Timms H. Understanding “New Power”. Harvard Business Review, 12; 2014 https://hbr.org/2014/12/understanding-new-power.

[52] Miller R, de Andrade M, Don RM. Culture & Values. In Amelung VE, Stein KV, Goodwin N, Balicer R, Nolte E, Suter E (eds.). Handbook Integrated Care. New York: Springer Nature; 2017 DOI: 10.1007/978-3-319-56103-5_15

[53] Zonneveld N, Driessen N, Stüssgen RA, Minkman MM. Values of Integrated Care: A Systematic Review. Int J Integr Care, 2018 Oct-Dec; 18(4): 9 DOI: 10.5334/ijic.4172 PMC6251066 30498405

[54] WHO Europe. Integrated care models: an overview Copenhagen: WHO Regional Office for Europe; 2016 http://www.euro.who.int/__data/assets/pdf_file/0005/322475/Integrated-care-models-overview.pdf?ua=1.

[55] Goodwin N, Stein V, Amelung V. What is integrated care? In Amelung VE, Stein KV, Goodwin N, Balicer R, Nolte E, Suter E (eds.). Handbook Integrated Care. New York: Springer Nature; 2017 DOI: 10.1007/978-3-319-56103-5_1

[56] Liang Z, Howard PF, Leggat S, Bartram T. Development and validation of health service management competencies. Journal of health organization and management, 2018 4 9; 32(2): 157–75. DOI: 10.1108/JHOM-06-2017-0120 29624143

[57] Pihlainen V, Kivinen T, Lammintakanen J. Management and leadership competence in hospitals: a systematic literature review. Leadership in Health Services, 2016 2 1; 29(1): 95–110. DOI: 10.1108/LHS-11-2014-0072 26764963

[58] Lega F, Prenestini A, Spurgeon P. Is Management Essential to Improving the Performance and Sustainability of Health Care Systems and Organizations? A Systematic Review and a Roadmap for Future Studies. Value in Health, 2016; 13: 46–51. DOI: 10.1016/j.jval.2012.10.004 23317645

[59] Goodwin N. Are networks the answer to achieving integrated care? J Health Serv Res Policy, 2008; 13(2): 58–60. DOI: 10.1258/jhsrp.2008.008001 18416908

[60] Miller R, Brown H, Mangan C. Integrated care in action: A practical guide for health, social care and housing support Jessica Kingsley Publishers; 2016 6 21.

[61] Lowndes V, Skelcher C. The dynamics of multi-organizational partnerships: an analysis of changing modes of governance. Public administration, 1998; 76(2): 313–33. DOI: 10.1111/1467-9299.00103

[62] Lecy JD, Mergel IA, Schmitz HP. Networks in public administration: current scholarship in review. Public Management Review, 2014 7 4; 16(5): 643–65. DOI: 10.1080/14719037.2012.743577

[63] Addicott R, McGivern G, Ferlie E. Networks, organizational learning and knowledge management: NHS cancer networks. Public Money and Management, 2006 4 1; 26(2): 87–94. DOI: 10.1111/j.1467-9302.2006.00506.x

[64] Cristofoli D, Meneguzzo M, Riccucci N. Collaborative administration: the management of successful networks. Public Management Review, 2017: 275–283. DOI: 10.1080/14719037.2016.1209236

[65] Sheaff R, Schofield J, Charles N, Benson L, Mannion R, Reeves D. The management and effectiveness of professional and clinical networks; 2011 http://www.netscc.ac.uk/hsdr/files/project/SDO_FR_08-1518-104_V01.pdf.

[66] Mintzberg H. The Nature of Managerial Work New York, NY: Harper & Row; 1973.

[67] Bartelings JA, Goedee J, Raab J, Bijl R. The nature of orchestrational work. Public management review, 2017 3 16; 19(3): 342–60. DOI: 10.1080/14719037.2016.1209233

[68] Willem A, Lucidarme S. Pitfalls and challenges for trust and effectiveness in collaborative networks. Public Management Review, 2014 7 4; 16(5): 733–60. DOI: 10.1080/14719037.2012.744426

[69] Randall S. Leading networks in healthcare London, United Kingdom: The Health Foundation Learning Report; 2013.

[70] Sandfort J, Milward HB. Collaborative service provision in the public sector In Cropper S, Ebers M, Huxham C, Smith Ring P (eds.). The Oxford Textbook of Interorganizational Relations. New York: Oxford University Press; 2008: 147–174. DOI: 10.1093/oxfordhb/9780199282944.003.0006

