# Supplementary material for: The Odyssey of Integration: Is Management its Achilles’ Heel?
Source: Int J Integr Care. 2020 Feb 26;20(1):7. doi: 10.5334/ijic.5440 (PMC7047758; doi:10.5334/ijic.5440)
Supplement: Appendix 1. — Management and leadership competency interview guide. [file ijic-20-1-5440-s1.pdf]

Management Competences for Integrated Care: Interview schedule

1) What stakeholder perspective could you provide regarding the management of integrated care?

   Patients/People   Policy   Research   Practice/Clinical/Services

2) We think that there are two distinct management roles in the oversight of integrated care (based on Mintzberg 1975):

   Strategic Managers – decision makers, interaction with environment, setting policies, goals & frameworks, supervision of operational managers

   Operational Managers – implementers, mediators between strategic and core, limited interaction with environment related to their delivery areas, departmental strategies within strategic framework

   Is this conceptualisation helpful / relevant?

3) From your experience, what (if anything) is different about strategic and operational management for integrated care in contrast to usual care?

4) Examples

   Can you provide an example of how strategic and/or operational management has been a strong enabler of integrated care?
   Can you provide an example of how strategic and/or operational management has been a barrier of integrated care?

5) What do you think is necessary (and different) about being a strategic and / or operational manager for integrated care in respect of –

a. Knowledge
b. Values
c. Skills

6) How can the organisational / partnership / policy context support / allow / prevent managers from demonstrating these behaviours?

7) Development

a. What fostered your development of these skills, values and knowledge?
b. Looking back, what would have helped you to develop them quicker / more thoroughly?
c. What organisational and partnership contexts enabled / prevented you to demonstrate these management competencies?

Anything else you would like to share?
